# The oncogenic E3 ligase TRIP12 suppresses epithelial–mesenchymal transition (EMT) and mesenchymal traits through ZEB1/2

**DOI:** 10.1038/s41420-021-00479-z

**Published:** 2021-05-07

**Authors:** Kwok Kin Lee, Deepa Rajagopalan, Shreshtha Sailesh Bhatia, Roberto Tirado-Magallanes, Wee Joo Chng, Sudhakar Jha

**Affiliations:** 1 Cancer Science Institute of Singapore, National University of Singapore, Singapore, 117599 Singapore; 2 Department of Biochemistry, Yong Loo Lin School of Medicine, National University of Singapore, Singapore, Singapore; 3 Department of Medicine, Yong Loo Lin School of Medicine, National University of Singapore, Singapore, 117596 Singapore; 4 Department of Haematology-Oncology, National University Cancer Institute of Singapore, National University Health System, Singapore, Singapore

**Keywords:** Breast cancer, Metastasis, Cell adhesion, Cell death, Cell polarity

## Abstract

Thyroid hormone receptor interactor 12 (TRIP12) is an E3 ligase most notably involved in the proteolytic degradation of the tumor suppressor p14ARF. Through this process, it is proposed that TRIP12 plays an oncogenic role in tumor initiation and growth. However, its role in other cancer processes is unknown. In this study, using publicly available cancer patient datasets, we found TRIP12 to be associated with distant metastasis-free survival in breast cancer, suggesting an inhibitory role in metastasis. Following TRIP12 depletion, an epithelial-mesenchymal transition (EMT) shift occurred with concomitant changes in EMT cell adhesion markers identified through RNA-seq. In line with EMT changes, TRIP12-depleted cells gained mesenchymal traits such as loss of cell polarity, dislodgement from bulk cells at a higher frequency, and increased cellular motility. Furthermore, ectopic TRIP12 expression sensitized cells to anoikis. Mechanistically, TRIP12 suppresses EMT through inhibiting ZEB1/2 gene expression, and ZEB1/2 depletion rescues EMT markers and mesenchymal behavior. Overall, our study delineates TRIP12’s role in inhibition of EMT and implies a potential suppressive role in breast cancer metastasis.

## Introduction

Thyroid hormone receptor interactor 12 (TRIP12) is a homologous to E6AP C terminus (HECT) domain E3 ubiquitin ligase essential in various cellular processes and pathways. It is required for embryonic development, as mouse embryos with TRIP12 homozygous mutation are embryonic lethal and have delayed development at E8.5 (ref. ^1^). Additionally, TRIP12 may regulate pancreatic cell homeostasis by regulating the protein stability of pancreas transcription factor 1a (PTF1A) (ref. ^2^). In the DNA damage context, TRIP12 prevents the uncontrolled spreading of chromatin ubiquitination through the degradation of ring finger protein 168 (RNF168), the E3 ligase responsible for histone ubiquitination upon DNA damage and subsequent recruitment of 53BP1 (ref. ^3^). In human papillomavirus-induced head and neck cancers where p16 levels are elevated, negative regulation of TRIP12 level by p16 increases sensitivity to radiotherapy due to the loss of DNA repair pathway^4^. More recently, a study discovered that TRIP12 is a poly(ADP-ribose) (PAR)-dependent E3 ubiquitin ligase responsible for degradation of poly(ADP-ribose) polymerase 1 (PARP1), much like the pioneer PAR-dependent E3 ligase ring finger protein 146 (RNF146/IDUNA) (refs. ^5–7^), thus constraining PARP inhibitor efficiency^8^. Apart from the above-mentioned pathways, TRIP12 also serves as the E3 ligase for the ubiquitin fusion degradation (UFD) pathway^9^, responsible for the degradation of substrates with an N-terminal linked ubiquitin^10^ and has been suggested to be the core degradation pathway for lysine-less substrates such as p14ARF (ref. ^11^). In addition, two studies show that TRIP12 is important for the maintenance of protein complex stoichiometry namely, the SWI/SNF complex and the APP-BP1 neddylation complex through the degradation of their unbound subunits BAF57 and APP-BP1 respectively^12,13^.

In the cancer context, TRIP12 is a key regulatory player in oncogene-induced senescence (OIS), a major barrier against cancer initiation. In normal human fibroblast cells, the steady-state level of p14ARF is tightly controlled through protein degradation mediated by TRIP12 (ref. ^14^). In oncogene-induced scenarios such as c-Myc amplification, this TRIP12-p14ARF regulation is disrupted, resulting in an increase of p14ARF level, thereby leading to OIS and the prevention of carcinogenesis^14,15^. In addition, several p14ARF interacting partners such as nucleophosmin (NPM), tumor necrosis factor receptor-associated death domain (TRADD), N-Myc and STATs interactor (NMI), and nucleostemin have also been reported to suppress cell growth or tumor formation through the stabilization of p14ARF by disrupting TRIP12-p14ARF degradation in different cell types including, normal fibroblast, acute myeloid leukemia, lung cancer cell lines, and in a mouse cancer model^14–19^. More recently, studies in liver cancer found that the deubiquitinating enzyme USP7 stabilizes TRIP12, which leads to constitutive p14ARF ubiquitination and degradation, thereby promoting the growth of liver cancer cells^20^. Interestingly, another study found that TRIP12 could degrade USP7 (ref. ^21^), suggesting a possible regulatory loop between TRIP12 and USP7. TRIP12 has also been found to have the highest frequency of mutations in lung adenocarcinoma patients, while frameshift mutations of TRIP12 are present in colorectal and gastric cancers with microsatellite instability^22,23^. Taken together, there is a consensus in the possible tumor-promoting role of TRIP12 through its proteolytic effect on p14ARF.

Although the role of TRIP12 as a potential promoter of cancer initiation has been studied, its role in various cancer types and in other cancer processes remains unknown. In this present study, we identified an inhibitory role for TRIP12 in suppressing epithelial-mesenchymal transition (EMT) and mesenchymal traits through regulating ZEB1/2 gene expression, contrary to its tumor-promoting role in cancer initiation.

## Results

### TRIP12 expression in breast cancer patients correlates with distant metastasis-free survival

**Fig. 1:**
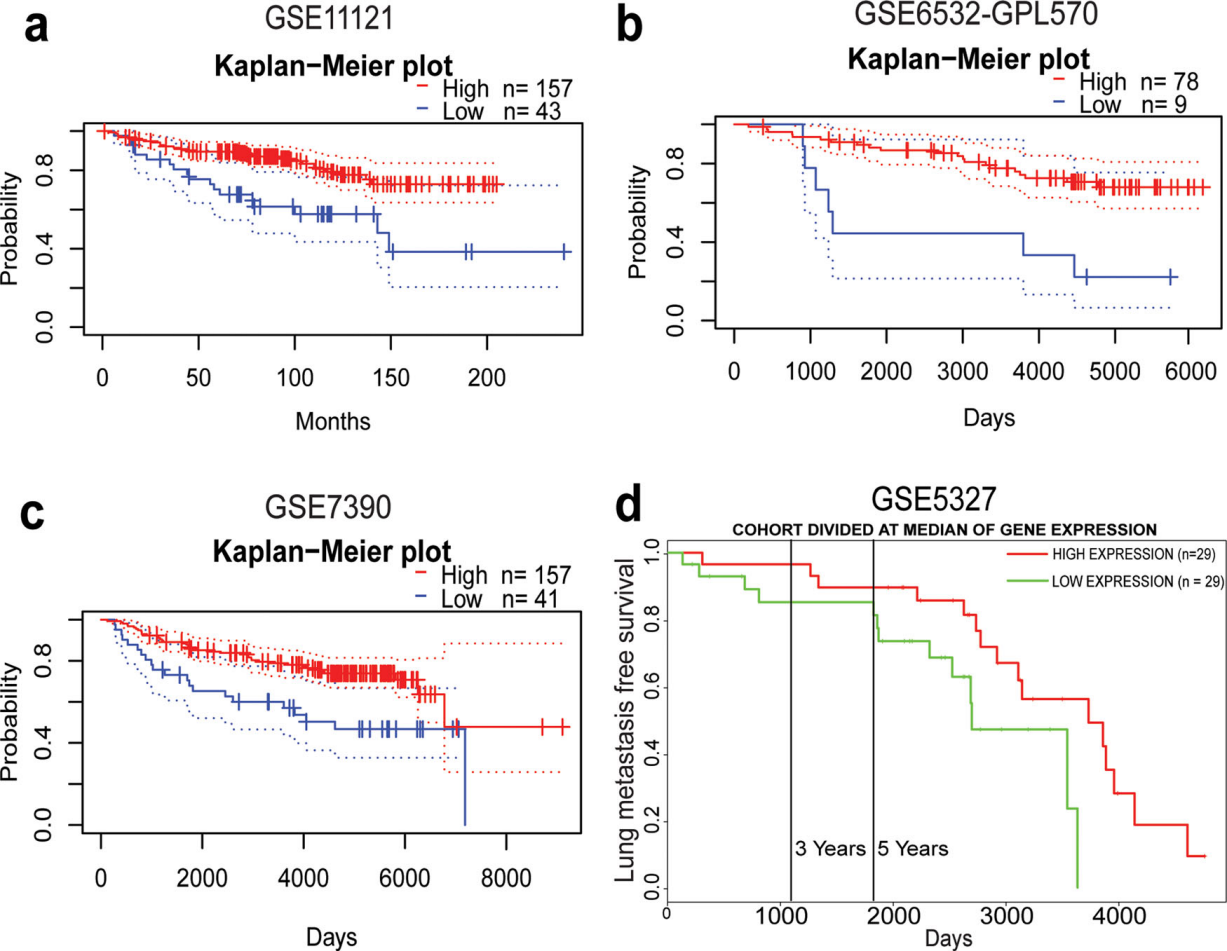
*TRIP12* expression in breast cancer patients correlates with distant metastasis-free survival. Kaplan–Meier distant metastasis-free survival curve for breast cancer patients in (**a**) GSE11121, **b** GSE6532-GPL570, **c** GSE7390, and (**d**) Kaplan–Meier lung metastasis-free survival curve for breast cancer patients in GSE5327.

As a preliminary step to study the role of TRIP12 in different cancers, online bioinformatics tools for meta-analysis were used to determine the clinical relevance of TRIP12 expression. Among different clinical parameters and different cancers, we observe that level of TRIP12 is positively associated with distant metastasis-free survival of breast cancer patients (Fig. 1). Through the PrognoScan tool^24^, TRIP12 correlates with distant metastasis-free survival in three publicly available breast cancer datasets. Distant metastasis-free survival is significantly lower (COX *p* value = 0.01819; HR = 0.20(95% CI: 0.05–0.76)) in breast cancer patients with low TRIP12 as compared to high TRIP12 levels in the GSE11121 study (Fig. 1a). Similarly, in GSE6532-GPL570, there is a significantly lower (COX *p* value = 0.03578; HR = 0.27(95% CI: 0.08–0.92)) probability of distant metastasis in high TRIP12 breast tumors as compared to low TRIP12 tumors (Fig. 1b). Lastly, distant metastasis-free survival is lower (*p* value = 0.03563; COX *p* value = 0.26621; HR = 0.70(95% CI: 0.37–1.32)) in TRIP12 low tumors than in TRIP12 high tumors in the GSE7390 breast cancer study (Fig. 1c). A similar correlation is observed in the GSE5327 breast cancer dataset, obtained through the PROGgeneV2 online database^25^, which shows that lung metastasis-free survival is lower (*p* value = 0.00337; HR = 0.21(95% confidence interval (CI): 0.07–0.60)) in tumors with low TRIP12 as compared to tumors with high TRIP12 levels (Fig. 1d). These data suggest that TRIP12 could have an inhibitory role in breast cancer distant metastasis.

### RNA-seq reveals cell adhesion molecules as the most significant pathway regulated by TRIP12

**Fig. 2:**
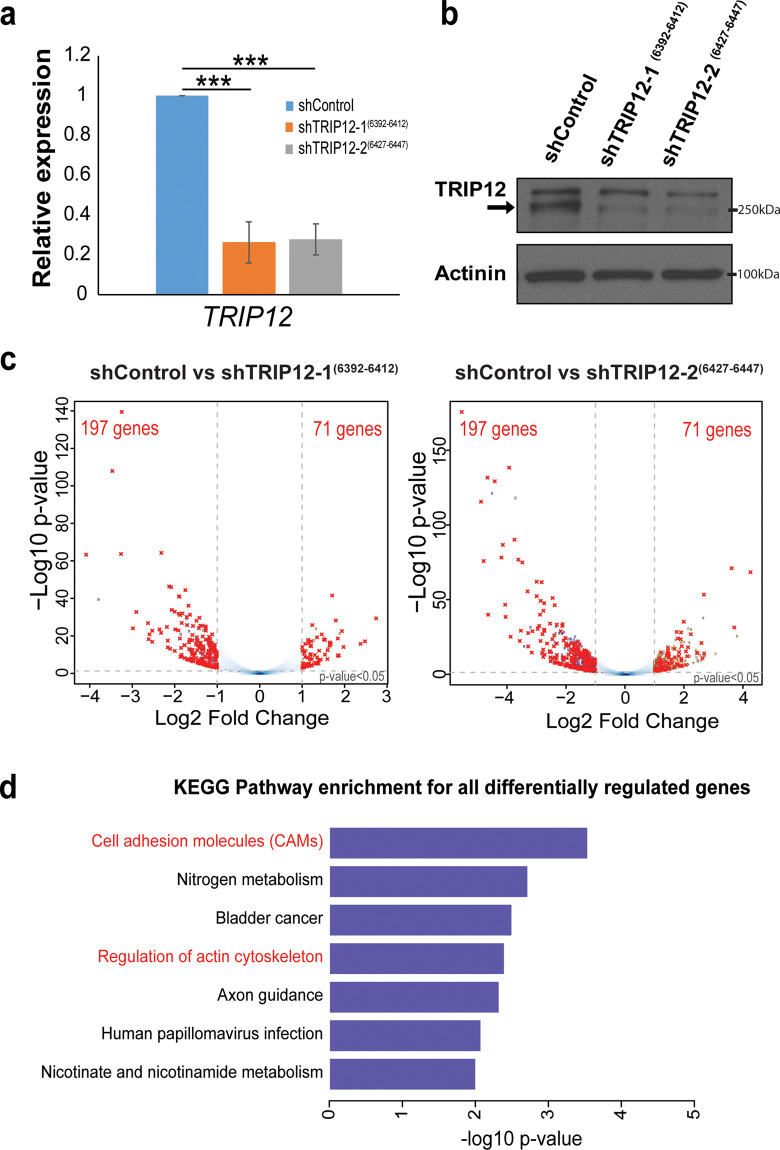
RNA-seq reveals cell adhesion molecules as the most significant pathway regulated by TRIP12. **a** Relative expression of *TRIP12* mRNA in MCF10A cells stably depleted of TRIP12. Relative expression levels were normalized to Actin mRNA levels and data quantified relative to shControl. (*N* = 3). Data represents means ± SD. ***p value < 0.001. **b** TRIP12 and Actinin protein levels in MCF10A cells stably depleted of TRIP12. Actinin serves as a loading control. Arrow indicates the TRIP12 band. (*N* = 3). **c** Volcano plots showing the differentially regulated genes upon TRIP12 depletion in MCF10A for shTRIP12-1^(6392–6412)^ (left panel) and shTRIP12-2^(6427–6447)^ (right panel), as compared to shControl. Differentially regulated genes were identified using the criteria: FDR < 0.05 and |Log2 fold change| >1. Dots marked in red represent the overlap of differentially regulated genes between shTRIP12-1^(6392–6412)^ and shTRIP12-2^(6427–6447)^. (*N* = 2 for each shRNA). **d** KEGG pathway analysis for all differentially regulated genes which overlap between shTRIP12-1^(6392–6412)^ and shTRIP12-2^(6427–6447)^ shown in (**c**). Pathways related to EMT are highlighted in red.

To study the role of TRIP12 in the breast context, the breast epithelial MCF10A cell model, which has been used to investigate the EMT process^26,27^ is utilized. Two stable cell lines expressing different shRNAs targeting TRIP12 were generated. The stable cell lines show a robust decrease in TRIP12 expression at the RNA (Fig. 2a) and protein (Fig. 2b) levels. EMT is a form of cellular reprograming characterized by wide-spread transcriptional changes in the gene expression of multiple EMT markers^28^. In order to characterize global gene expression changes regulated by TRIP12, RNA-sequencing (RNA-seq) was performed on the generated stable cells depleted of TRIP12. Genes that are significantly differentially expressed (FDR < 0.05 and |log2 (fold change)| >1) in the two stable cell lines with different shRNA targeting TRIP12 are represented by volcano plots in Fig. 2c. The list of all differentially expressed gene from the two stable cell lines are shown in Supplementary Table 1. Overlap of the significantly differentially regulated genes between the two stable cell lines identified 197 downregulated genes and 71 upregulated genes (Fig. 2c) regulated by TRIP12.

Pathway analysis of the overlapped differentially regulated genes enriches for several pathways, including “cell adhesion molecules (CAMs)”, “nitrogen metabolism”, “bladder cancer”, “regulation of actin cytoskeleton”, “axon guidance”, “human papillomavirus infection”, and “nicotinate and nicotinamide metabolism” (Fig. 2d). Among the pathways regulated, “cell adhesion molecules (CAMs)” and “regulation of actin cytoskeleton” represent EMT-related processes, with “cell adhesion molecules (CAMs)” as the most significantly enriched pathway (Fig. 2d). Cell adhesion molecules play an important role in maintaining cell–cell contact and are frequently altered during epithelial–mesenchymal transition (EMT)^29–33^. Examples include the epithelial marker E-cadherin (*CDH1*) and mesenchymal marker N-cadherin (*CDH2*), which are canonical EMT markers. Since TRIP12 affects the expression of cell adhesion molecules, we decided to study the alteration in cell adhesion molecules expression by TRIP12 and its effects.

### TRIP12 regulates EMT cell adhesion genes involved in adherens junctions, tight junctions, desmosomes, cytoskeleton, and extracellular matrix interaction

**Fig. 3:**
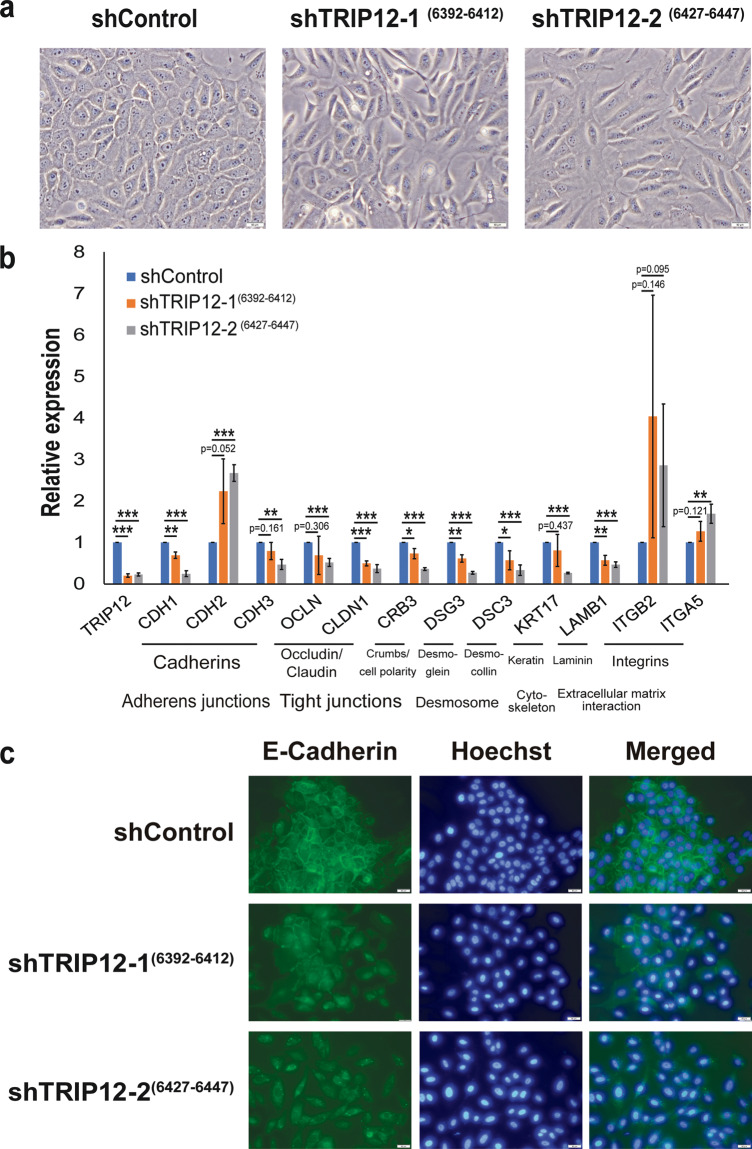
TRIP12 regulates EMT cell adhesion genes involved in adherens junctions, tight junctions, desmosomes, cytoskeleton, and extracellular matrix interaction. **a** Bright-field images showing the cellular morphology of MCF10A shControl, shTRIP12-1^(6392–6412)^ and shTRIP12-2^(6427–6447)^. Images were taken at 20X magnification. The scale bar at the bottom right corner = 50 µm. (*N* = 1). **b** Relative expression of the mRNA levels of EMT markers in MCF10A cells stably depleted of TRIP12. Relative expression levels were normalized to Actin mRNA levels and data quantified relative to shControl. (*N* = 3). Data represent means ± SD. *p value < 0.05; ** *p* value < 0.01; *** *p* value < 0.001. **c** Fluorescent images of MCF10A shControl, shTRIP12-1^(6392–6412)^ and shTRIP12-2^(6427–6447)^ immunostained using an E-cadherin antibody and with Hoechst dye. Images were taken at 20X magnification. The scale bar at the bottom right corner = 50 µm. (*N* = 1).

Corroborating with changes in cell adhesion molecules gene expression, morphological changes are observed in MCF10A cells depleted of TRIP12 (Fig. 3a). Cells lose their cobblestone morphology, are more elongated, and exhibit a “fibroblast-like” spindle morphology (Fig. 3a), typical of cells that have undergone EMT. Images were taken at a high density because MCF10A cell density has been reported to affect morphology, whereby cells display an epithelial morphology at high density while adopting a mesenchymal morphology at low density^26,27^. TRIP12-depleted cells at high density still show a mesenchymal morphology (Fig. 3a), confirming that these morphological changes are not due to cell density.

Next, quantitative-Polymerase Chain Reaction (qPCR) was utilized to validate genes involved in cell adhesion. TRIP12 downregulation alters the expression of different cellular junction genes (Fig. 3b). Notably, changes in adherens junctions include the loss of epithelial markers E-cadherin (*CDH1*) and P-cadherin (*CDH3*), and the gain of mesenchymal marker N-cadherin (*CDH2*) (Fig. 3b). This is typically termed “cadherin switching” and is observed in EMT during embryonic development^34^. In addition, TRIP12-depleted cells show a decrease in membrane E-cadherin protein (Fig. 3c), corroborating with changes in E-cadherin gene expression. Tight junction structural proteins occludin (*OCLN*) and claudin 1 (*CLDN1*), and tight junction-associated protein crumbs cell polarity complex component 3 (*CRB3*) are also downregulated upon TRIP12 depletion (Fig. 3b). Desmosomes are a component of the epithelial junctional complex and aid in cell-cell adhesion^35^. Structural components of the desmosome, desmocollin-3 (*DSC3*), and desmoglein-3 (*DSG3*) show a decrease upon TRIP12 depletion (Fig. 3b). Other important signaling and EMT-related molecules such as keratin (*KRT17*), laminin (*LAMB1*), and integrins (*ITGB2* and *ITGA5*) are also altered upon TRIP12 depletion (Fig. 3b). In addition, changes in EMT markers are also validated in two separate breast cancer cell lines (Supplementary Fig. 1). Depletion of TRIP12 in MDA-MB-468 resulted in decrease in epithelial markers *OCLN*, *CLDN1*, and *CRB3* (Supplementary Fig. 1a). TRIP12 knockdown in CAL51 leads to a reduction in EMT markers, *CRB3* and *DSG3* (Supplementary Fig. 1b). These results validate the RNA-seq data in which TRIP12 depletion leads to major gene expression changes in cell adhesion molecules.

### Alterations in TRIP12 level results in loss of cellular polarity, increase in single-cell dislodgment, increase in cell motility, and sensitivity to anoikis

**Fig. 4:**
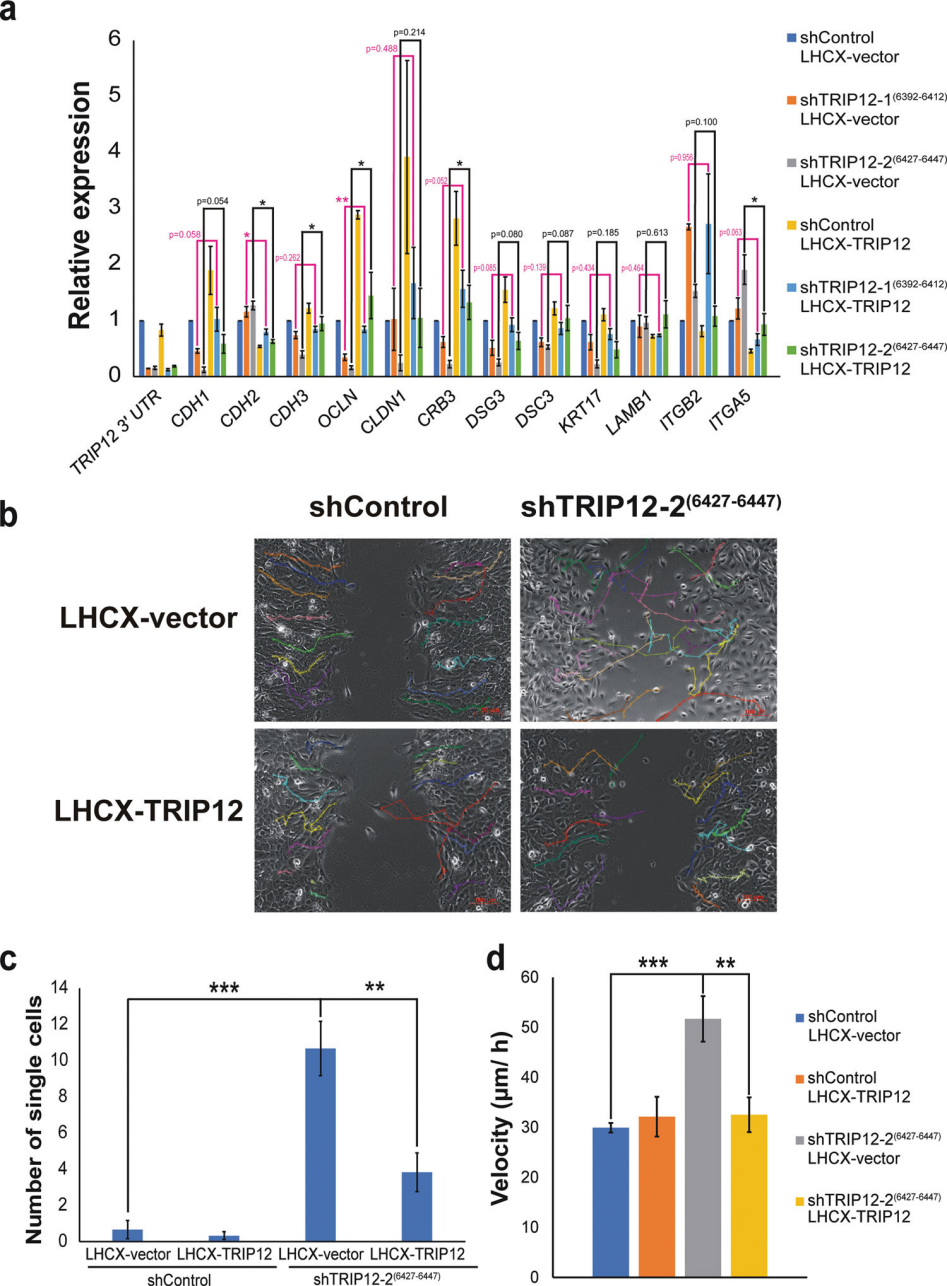
Alterations in TRIP12 level results in loss of cellular polarity, increase in single-cell dislodgment, increase in cell motility, and sensitivity to anoikis. **a** Relative expression of the mRNA levels of EMT markers in MCF10A cells stably depleted of TRIP12 and with LHCX-vector expression or LHCX-TRIP12 expression. Relative expression levels were normalized to Actin mRNA levels and data quantified relative to shControl LHCX vector. (*N* = 3). Data represent means ± SEM. * *p* value < 0.05; ** *p* value < 0.01. **b** Representative cell tracking snapshot images of cell migration/ wound-healing assay for MCF10A shControl and shTRIP12-2^(6427–6447)^, with either LHCX-vector or LHCX-TRIP12 expression, at midpoint to wound closure. Fifteen single cell tracks are represented with different colors in each image. Images were taken at 10X magnification. The scale bar at bottom right corner = 100 µm. **c** Quantification of the number of single cells in the remaining wound area from images when the remaining wound area is 35–55% of the total area. (*N* = 6). Data represent means ± SEM. ** *p* value < 0.01; *** *p* value < 0.001. **d** Velocity of tracked MCF10A shControl and shTRIP12-2^(6427–6447)^, with either LHCX-vector or LHCX-TRIP12 expression. Fifteen cell tracks per condition were used for acquiring velocity data. Data represent means ± SEM. ** *p* value < 0.01; *** *p* value < 0.001.

To study if the expression changes in cell adhesion molecules are specific to TRIP12, stable cell lines with ectopic expression of TRIP12 are created. The cell lines with ectopic TRIP12 expression show an increase in TRIP12 RNA level, which can be differentiated from endogenous TRIP12 using qPCR primers either targeting TRIP12 3’UTR or CDS (Fig. 4a, Supplementary Fig. 2a). Rescue in TRIP12 level is observed at both RNA (Supplementary Fig. 2a) and protein (Supplementary Fig. 2b) levels in the stable cell lines expressing ectopic TRIP12 (Supplementary Fig. 2). Upon ectopic TRIP12 expression, most cell adhesion gene expression changes are partially rescued (Fig. 4a). Epithelial markers *CDH1*, *CDH3*, *OCLN*, *CLDN1*, *CRB3*, *DSG3*, *DSC3*, and *KRT17*, show a higher level in cells with TRIP12 depletion and ectopic LHCX-TRIP12 expression, as compared to cells with TRIP12 depletion and ectopic LHCX-vector expression (Fig. 4a). Among these, the level of *OCLN*, *CDH3* (for shTRIP12-2^(6427–6447)^ LHCX-TRIP12) and *CRB3* (for shTRIP12-2^(6427–6447)^ LHCX-TRIP12), are significantly rescued.

Mesenchymal markers *CDH2*, *ITGB2* (for shTRIP12-2^(6427–6447)^ LHCX-TRIP12) and *ITGA5*, show a lower level in cells with TRIP12 depletion and ectopic LHCX-TRIP12 expression, as compared to cells with TRIP12 depletion and LHCX-vector expression (Fig. 4a). Specifically, mesenchymal markers *CDH2* and *ITGA5* (for shTRIP12-2^(6427–6447)^ LHCX TRIP12) show a significant rescue in level. Notably, ectopic expression of TRIP12 in shControl cells also results in an opposite trend to TRIP12 depletion for both epithelial and mesenchymal markers (Fig. 4a), affirming the role of TRIP12 in regulating cell adhesion genes, and depicting the plasticity of MCF10A to traverse along the EMT spectrum.

To investigate if TRIP12 has a role to play in EMT-related processes, a wound-healing assay was performed in MCF10A shTRIP12-2^(6427–6447)^ cells, which show a larger extent of change in EMT markers expression as compared to MCF10A shTRIP12-1^(6392–6412)^ cells (Fig. 3a). Single-cell tracking was employed to detect cell movement and quantify cell motility. Fifteen cells from each condition were tracked. Movies of wound-healing assays without tracking and with tracking are represented in Supplementary movies 1–4 and 5–8 respectively. Snapshots of tracks at midpoint to wound closure are shown in Fig. 4b. From these results, we observe that the single-cell tracks of shControl LHCX-vector are ordered and neat, with cells moving unidirectionally towards the wound area (Supplementary Movie 5 and Fig. 4b). The cells move as “sheets” and together with bulk cells as observed in Supplementary Movie 5, typical of epithelial cells. There are few to no cells that dislodge from the migrating bulk cells as quantified in Fig. 4c. The tracks do not cover the wound area, as shown in snapshots at midpoint to wound closure (Fig. 4b).

On the contrary, the tracks of shTRIP12-2^(6427–6447)^ LHCX-vector are chaotic and random, frequently moving back and forth without much direction, typical of cells that lose polarity (Supplementary movie 6 and Fig. 4b). In the snapshot at midpoint to wound closure (Fig. 4b), it can clearly be seen that the tracks occupy the wound area with frequent overlapping tracks, a stark contrast to shControl LHCX-vector where no tracks are observed in the wound area (Fig. 4b). Significantly more single cells separated from bulk cells than shControl LHCX-vector (Fig. 4c). In addition, we quantified the velocity of the tracked cells. shTRIP12-2^(6427–6447)^ LHCX-vector cells exhibited a significantly higher velocity as compared to shControl LHCX-vector cells, indicating a gain in cell motility upon TRIP12 depletion (Fig. 4d).

Remarkably, in the shTRIP12-2^(6427–6447)^ LHCX-TRIP12 cells where TRIP12 level is rescued, these mesenchymal traits are rescued. The tracks of shTRIP12-2^(6427–6447)^ LHCX-TRIP12 are ordered and move unidirectionally towards the wound area, together with bulk cells (Supplementary Movie 8 and Fig. 4b), similar to shControl LHCX-vector. In the snapshot at midpoint to wound closure (Fig. 4b), it can be observed that almost no tracks occupy the wound area (Fig. 4b), similar to shControl LHCX-vector and a stark contrast to shTRIP12-2^(6427–6447)^ LHCX-vector. There is also a significant rescue in the number of single cells dislodged from bulk cells (Fig. 4c). More importantly, cell motility, in terms of velocity, is rescued to shControl LHCX-vector level upon TRIP12 rescue, indicating that the cellular motility effect is specific and dependent on TRIP12 (Fig. 4d).

Anoikis is the induction of programmed cell death in the absence of cell attachment or attachment to inappropriate surfaces^36^. Resistance to anoikis is a feature acquired by metastatic cells to survive in circulation and successful colonization on distant organs^36^. Expression of *OCLN*, one of the cell adhesion genes regulated by TRIP12 (Fig. 4a), has been shown to decrease cancer cell’s resistance to anoikis^37^. Similarly, ectopic expression of TRIP12, which increases *OCLN* expression (Fig. 4a), results in a decrease in viability when cells are seeded on agarose-coated wells to induce anoikis (Supplementary Fig. 3a). Subsequently, decreasing the total TRIP12 level using shRNA results in a rescue in viability (Supplementary Fig. 3a), with corresponding *OCLN* expression level changes (Fig. 4a), indicating TRIP12’s specificity in anoikis. Additionally, this effect is specific to anoikis and not a case of general cell death due to TRIP12 ectopic expression, as supported by Supplementary Fig. 3b where there is no difference in cell viability when cells are seeded on tissue-culture plates and viability measured. These results depict TRIP12’s role in anoikis induction (Supplementary Fig. 3a).

### TRIP12 regulates EMT and metastasis-related processes through ZEB1/2 gene expression

**Fig. 5:**
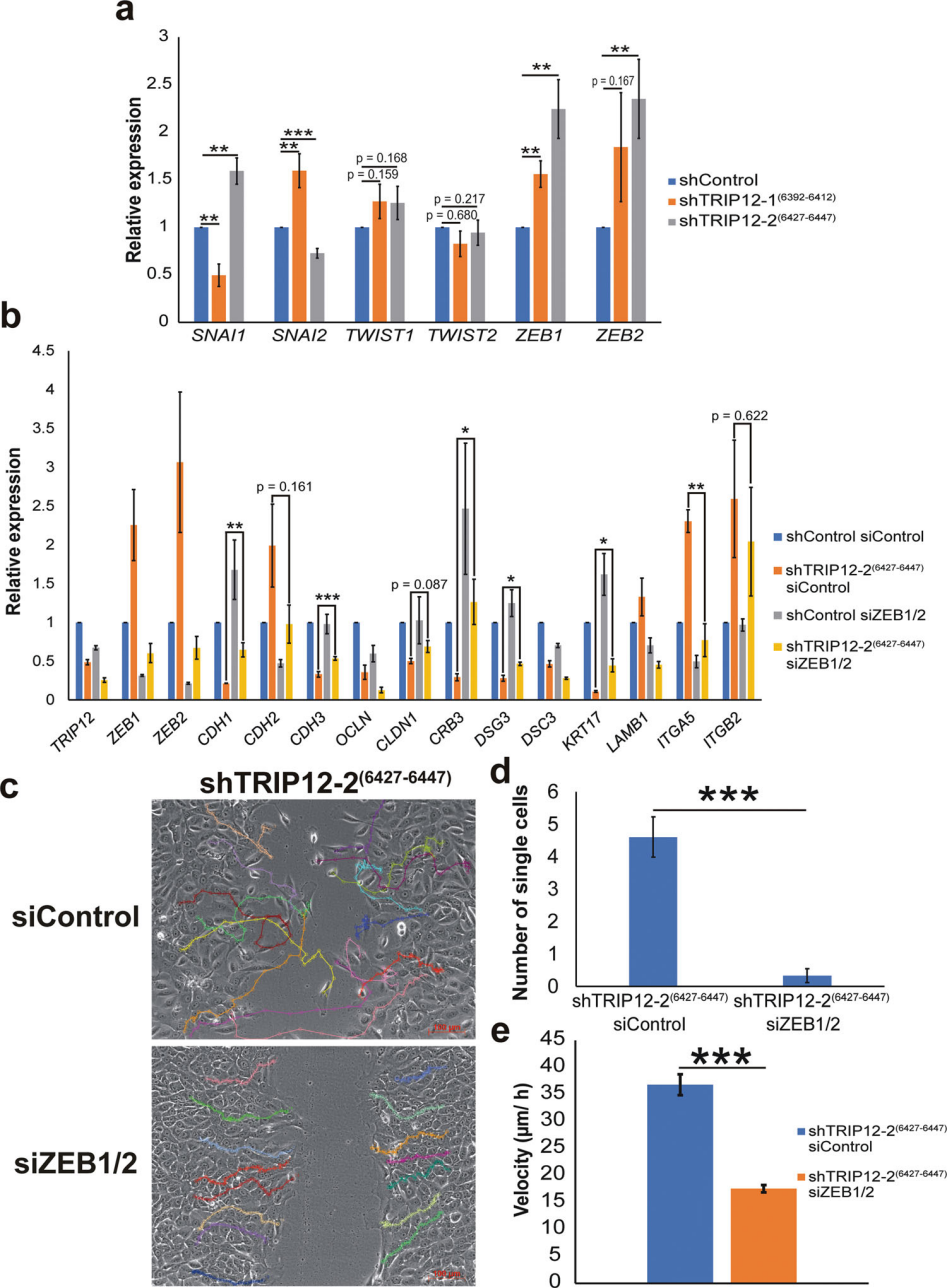
TRIP12 inhibits ZEB1/2 gene expression to regulate EMT and cellular behavior. **a** Relative expression of the mRNA levels of EMT transcription factors in MCF10A cells stably depleted of TRIP12. Relative expression levels were normalized to Actin mRNA levels and data quantified relative to shControl. (*N* = 7). Data represent means ± SEM. ** *p* value < 0.01; *** *p* value < 0.001. **b** Relative expression of the mRNA levels of EMT markers in MCF10A cells stably depleted of TRIP12 and with ZEB1/2 depletion. Relative expression levels were normalized to Actin mRNA levels and data quantified relative to shControl siControl. (*N* = 3). Data represent means ± SEM. * *p* value < 0.05; ** *p* value < 0.01; *** *p* value < 0.001. **c** Representative cell tracking snapshot images of cell migration/ wound-healing assay for MCF10A shTRIP12-2^(6427–6447)^, with either siControl or siZEB1/2, at midpoint to wound closure. Fifteen single cell tracks are represented with different colors in each image. Images were taken at 10X magnification. The scale bar at the bottom right corner = 100 µm. **d** Quantification of the number of single cells in the remaining wound area from images when the remaining wound area is 35–45% of the total area. (*N* = 5). Data represent means ± SEM. ***, *p* value < 0.001. **e** Velocity of tracked MCF10A shTRIP12-2^(6427–6447)^ siControl and siZEB1/2 cells. Fifteen cell tracks per condition were used for acquiring velocity data. Data represent means ± SEM. *** *p* value < 0.001.

The canonical EMT transcription factors (EMT-TFs), comprising of the snail family transcription repressors (SNAI1, SNAI2), the zinc finger E-box binding homeobox family (ZEB1, ZEB2), and the twist family bHLH transcription factor family (TWIST1), represent the molecular drivers of EMT^32,38^. These transcription factors drive EMT mainly through direct or indirect effects on different cellular junction proteins, such as the repression of *CDH1* and polarity factor *CRB3* (refs. ^32,38–45^). Among the EMT-TFs, RNA-seq data shows *ZEB1* and *ZEB2* gene expression significantly upregulated, with *ZEB2* expression increasing more than two-folds upon TRIP12 depletion (Supplementary Table 1). To validate, we performed gene expression analysis for EMT-TFs using qPCR. TWIST2 is also included as it has also been shown to promote EMT^46^. Following TRIP12 depletion, only *ZEB1* and *ZEB2* expression increases consistently in both shTRIP12-1^(6392–6412)^ and shTRIP12-2^(6427–6447)^ (Fig. 5a). No significant change was noted for *TWIST1* and *TWIST2* (Fig. 5a). On the contrary, *SNAI1* and *SNAI2* change in opposing directions between shTRIP12-1^(6392–6412)^ and shTRIP12-2^(6427–6447)^ (Fig. 5a). This could be due to differences in quasi-mesenchymal states within the spectrum that these two different shTRIP12 stable lines are in. Different combinations and timely contribution of different EMT-TFs are known to determine the eventual EMT state^31,47^. This is further supported by the degree of change of the EMT markers between shTRIP12-1^(6392–6412)^ and shTRIP12-2^(6427–6447)^, which indicates that shTRIP12-2^(6427–6447)^ is in a further mesenchymal state in the EMT spectrum as compared to shTRIP12-1^(6392–6412)^ (Fig. 3b). Additionally, the degree of change in EMT markers (Fig. 3b) correlates with the degree of change of ZEB1 and ZEB2 in shTRIP12-1^(6392–6412)^ and shTRIP12-2^(6427–6447)^ (Fig. 4a), suggesting that ZEB1/2 expression regulation by TRIP12 (Fig. 5a) regulates TRIP12-dependent EMT inhibition.

To study the role of ZEB1 and ZEB2 in TRIP12-dependent EMT inhibition, a co-depletion of *ZEB1* and *ZEB2* using siRNA was performed. Starkly, depletion of both *ZEB1* and *ZEB2* rescued the expression of epithelial genes *CDH1*, *CDH3*, *CLDN1*, *CRB3*, *DSG3*, and *KRT17* (Fig. 5b). Among these, *CDH1*, *CDH3*, *CRB3*, *DSG3*, and *KRT17* gene expression are significantly rescued (Fig. 5b). On the other hand, mesenchymal genes *CDH2*, *ITGA5*, and *ITGB2* are rescued upon co-depletion of *ZEB1* and *ZEB2*, with *ITGA5* gene expression significantly rescued (Fig. 5b). This shows that TRIP12 regulates these EMT cell adhesion genes by suppressing *ZEB1* and *ZEB2* expression. Additionally, *OCLN*, *DSC3*, and *LAMB1* were not rescued upon ZEB1/2 depletion (Fig. 5b). This suggests that there could be ZEB1/2 independent mechanisms which TRIP12 could regulate *OCLN* and *DSC3* as these genes are still rescued with ectopic TRIP12 (Fig. 4a). The expression of *LAMB1* is an example of a non-robust target, which also does not decrease robustly in Fig. 4a upon TRIP12 depletion, and acts as a negative control (Fig. 5b).

Next, we performed wound-healing assay to investigate the effect of ZEB1/2 depletion on TRIP12-dependent EMT inhibition. Again, we employ single-cell tracking to detect cell movement and measure cell motility. Fifteen cells from each condition were tracked. Movies without cell tracking and with cell tracking are represented in Supplementary Movies 9–10 and 11–12, respectively. Snapshots of the tracks at midpoint to wound closure are shown in Fig. 5c. Upon ZEB1/2 depletion, the tracks of shTRIP12-2^(6427–6447)^ siZEB1/2 restored to an ordered and neat manner compared to the complex pattern observed in shTRIP12-2^(6427–6447)^ siControl, indicating a rescue in mesenchymal phenotype (Fig. 5c). Also, we observe that upon ZEB1/2 depletion, the tracks are unidirectional moving towards the wound area, as compared to the tracks of shTRIP12-2^(6427–6447)^ siControl which are messy and moves in different directions (Supplementary Movies 11–12 and Fig. 5c). Depletion of ZEB1/2 also resulted in no tracks seen in the wound area at midpoint to wound closure, contrasting with siControl (Fig. 5c). Significantly fewer single cells dislodged from bulk cells upon ZEB1/2 depletion (Fig. 5d). More importantly, cell motility, in terms of velocity, decreases significantly upon ZEB1/2 depletion (Fig. 5e). Overall, this data confirm that TRIP12-dependent EMT phenotypes are dependent on ZEB1/2.

## Discussion

In physiologically normal conditions, TRIP12 targets the tumor suppressor p14ARF for degradation to maintain it at a steady-state level^14^. However, this equilibrium changes when oncogenic stress (for example, c-Myc amplification) inhibits the degradation of p14ARF by TRIP12, thereby increasing p14ARF levels and inducing OIS^14,15^. OIS plays a crucial role in preventing cells from turning cancerous and as such is an important player in prohibiting early-stage carcinogenesis^48^. Based on these findings, changes in TRIP12 level is likely to play an oncogenic role in carcinogenesis and it has been proposed that TRIP12 could serve as a therapeutic target in specific scenarios i.e. when TRIP12 is dysregulated^11^ or specifically in acute myeloid leukemia where nucleophosmin is attenuated^16^. However, our study illustrates a novel role for TRIP12 in suppressing EMT and mesenchymal traits. Specifically, loss of TRIP12 induces an EMT (Fig. 3) and results in gain of mesenchymal traits, such as loss of cell polarity, increase in single-cell dislodgment, and increase in cellular motility (Fig. 4b-d, and Supplementary movies 1–8). Conversely, TRIP12 overexpression sensitizes cells to anoikis (Supplementary fig. 3). Additionally, the effects of EMT and mesenchymal phenotypes are dependent on ZEB1/2 gene expression (Fig. 5). These results are in line with patient datasets which show a correlation of TRIP12 with distant metastasis-free survival (Fig. 1). These results depict TRIP12’s suppressive role in EMT and imply a potential inhibitory role in cancer metastasis.

A plausible reason for the disparity in TRIP12’s function between this study and Chen et al (2010a) could be due to the genetic background of the cell model MCF10A used in this study. The immortal MCF10A cell line, which arose spontaneously from mortal mammary epithelial cells, harbors a reciprocal translocation t (3;9) (p14;p21), resulting in the deletion of the *CDKN2A* locus containing p16 and p14ARF (refs. ^49–51^). The inactivation of the *CDKN2A* locus has also been reported in other immortalized mammary cell lines, illustrating the importance of losing p16 and p14ARF, and in turn loss of OIS, in the immortalization process^52^. This suggests that while TRIP12 plays a cancer transformation promoting role in p14ARF positive normal fibroblast cells through the degradation of p14ARF resulting in loss of OIS, TRIP12 might play an EMT inhibitory role in p14ARF absent immortalized MCF10A cells, which have overcome OIS during the process of immortalization.

Although TRIP12 is an E3 ligase involved in protein degradation, this study finds that TRIP12 regulates the expression of multiple cell adhesion genes, mainly through inhibiting ZEB1/2 gene expression. Although the mechanism of how TRIP12 regulates ZEB1/2 expression is not addressed in this study, previous literature has shown that TRIP12 could regulate gene expression through the degradation of epigenetic factors such as the ARID1A complex component BAF57 (refs. ^1,13^) and the Polycomb repressive deubiquitinase (PR-DUB) complex components ASXL1 and BAP1, through a DNA N^6^-Methyladenine sensor network^53^. Whether TRIP12 regulates ZEB1/2 through these epigenetic regulators or other regulators requires further investigation.

Apart from cancer, recurrent TRIP12 mutations occur frequently in patients with neurodevelopmental diseases exhibiting symptoms of autism spectrum disorder, intellectual disability, and craniofacial dysmorphism^54–56^. However, a causal role for TRIP12 in this aspect has not been established. Our finding that TRIP12 regulates EMT could provide some clues on TRIP12’s role in this disease. EMT is an embryonic program required for many processes in embryonic development^32^. Specifically, primary EMT during nervous system development drives the formation of migratory neural crest cells, giving rise to peripheral nervous system cells, craniofacial structures, melanocytes, and some endocrine cells^32,57^. Taken together with our finding, TRIP12 mutations could be affecting EMT during nervous system development, thereby giving rise to neurodevelopmental diseases.

**Fig. 6:**
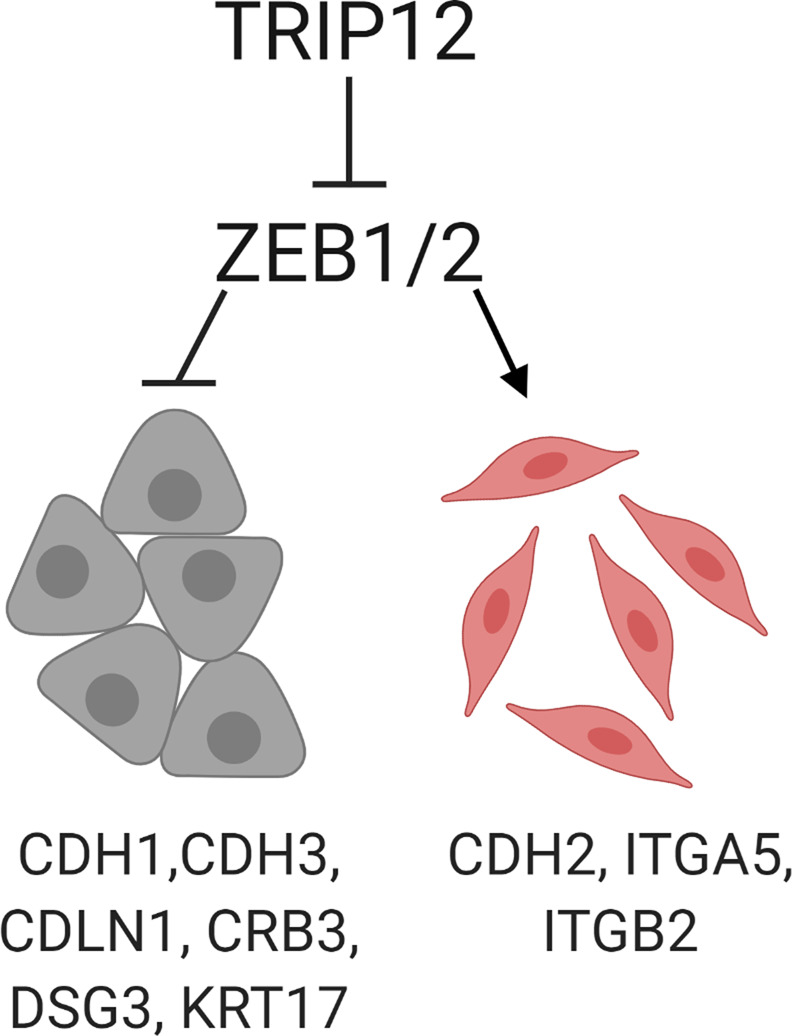
TRIP12 inhibits EMT through repressing ZEB1/2 regulated cell adhesion gene expression. Image was created using Biorender software.

In summary, we have identified a novel EMT and mesenchymal traits inhibitory role for TRIP12 through ZEB1/2 (Fig. 6). In our study, we found several lines of evidence both in the cellular context and in patients’ datasets, which support this newly found function of TRIP12.

## Materials and methods

### Cell culture

MCF10A (ATCC CRL-10317) was grown in Dulbecco’s modified eagle’s media (DMEM)/F12 (1:1) media (Gibco, Cat. No. 11330-032) with 5% horse serum (Gibco, Cat. No. 16050-122) and 1% penicillin–streptomycin (Gibco Cat. No. 15140-122). Additional additives include 20 ng/mL epithelial growth factor (Peprotech, Cat. No. AF-100-15), 0.5 mg/mL hydrocortisone (Sigma-Aldrich, Cat. No. H-0888), 100 ng/mL cholera toxin (Sigma-Aldrich, Cat. No. C-8052) and 10 μg/mL insulin (Sigma-Aldrich, Cat. No. I-1882). HEK293T (ATCC CRL-3216), MDA-MB-468 and CAL51 were grown in DMEM high glucose (Hyclone Cat. No. SH30243.01) with 10% fetal bovine serum (Sigma Cat. No. F-7524) and 1% penicillin-streptomycin (Gibco Cat. No. 15140-122). Cells were grown in a 5% CO_2_ incubator at 37 °C. Media was changed 1 day after cells were revived.

### Antibodies

Primary antibodies used were the following: TRIP12 (Bethyl Laboratories, Cat. No. A301-814A), α-Actinin (Santa Cruz, Cat. No. sc-17829), and E-cadherin/CDH1 antibody (BD Biosciences, Cat. No. 610182).

### shRNA

shRNA was cloned into pLKO.1 vector using restriction enzyme sites *Age*I (NEB, Cat. No. R3552) and *Eco*RI (NEB, Cat. No. R3103). pLKO.1 puro was a gift from Bob Weinberg (Addgene plasmid # 8453; http://n2t.net/addgene:8453; RRID: Addgene_8453). shRNA sequences used in this study are as follows:

**Table:** 

shRNA	Sequence
shControl	5'-ATGTTTACTACACTCGGATAT-3'
shTRIP12-1^(6392–6412)^	5'-ATGCTTTGCTGTGTGAAATTT-3'
shTRIP12-2^(6427–6447)^	5'-TTTCCAGGCTGGAACAATAAA-3'

### siRNA

siRNA used in this study are as follows:

**Table:** 

siRNA	Sequence
siControl	5'-CGUACGCGGAAUACUUCGA-3'
siZEB1	5'-CGGCGCAATAACGTTACAAAT-3'
siZEB2	5'-GGACACAGGUUCUGAAACA-3'

### siRNA transfection

siRNA was transfected using Lipofectamine RNAimax (Thermo Scientific, Cat. No. 13778150) according to the manufacturer’s protocol. Transfection mix was prepared by addition of 1 mL of Opti-MEM (Thermo Scientific, Cat. No. 31985070), 5 µL of Lipofectamine RNAimax and 20 nM total siRNA (for RNA analysis) or 10 nM total siRNA (for cell migration/ wound-healing assay) required for a total volume of 5 mL. The mixture was mixed and left standing for 30 min before adding 8 × 10^5^ cells in a 10-cm dish with a total volume of 5 mL (complete media with cells and transfection mix). After 6 h, fresh media was used to replace the transfection media. Cells were harvested after 48 h from the time of transfection.

### Plasmid

The pAcGFP-C1-TRIP12 plasmid was provided by Professor Jiri Lukas. TRIP12 CDS was subcloned into the LHCX vector (Clontech, Cat. No. 631511) using a single *Not*I site introduced into the LHCX vector through the *Hind*III site.

### Generation of stable cell lines

Virus was generated by transfecting 3 × 10^6^ HEK293T cells with retroviral LHCX (5 µg) or lentiviral pLKO.1 (3 µg) transfer plasmid using Lipofectamine 2000 (Thermo Scientific, Cat. No. 11668019) in a 10-cm tissue culture dish. Packaging and envelope plasmids were co-transfected with transfer plasmids. Lentiviral packaging plasmids used were pRSV-Rev (1.5 µg) and pMDLg/pRRE5 (1.5 µg). Lentiviral envelope plasmid used was pMD2.G (1.5 µg). pRSV-Rev was a gift from Didier Trono (Addgene plasmid #12253; RRID: Addgene_12253). pMDLg/pRRE was a gift from Didier Trono (Addgene plasmid #12251; RRID: Addgene_12251). pMD2.G was a gift from Didier Trono (Addgene plasmid #12259; RRID: Addgene_12259). Retroviral packaging and envelope plasmids used were pUMVC (3 µg) and pCMV-VSV-G (3 µg), respectively. pUMVC was a gift from Bob Weinberg (Addgene plasmid #8449; RRID: Addgene_8449). pCMV-VSV-G was a gift from Bob Weinberg (Addgene plasmid #8454; RRID: Addgene_8454). Viruses were harvested, filtered through 0.45 µm filter, and used to infect 5 × 10^5^ MCF10A cells (seeded 1 day before) with polybrene (Sigma-Aldrich, Cat. No. S2667) (4 µg/mL). After 24 h, the media was replaced with fresh media and left for 24 h for cell recovery. Media containing puromycin (Sigma, Cat. No. P9620) (1 µg/mL) (selection for lentiviral pLKO.1 plasmids) or hygromycin (Invitrogen, Cat. No. 10687010) (50 µg/mL) (selection for retroviral LHCX plasmids) was added and changed every 48 h till mock-transfected cells died. Cells were grown in antibiotics containing media for 2 weeks for the creation of stable cells.

### RNA extraction

RNA was extracted using TRIZOL reagent (Life Technologies, Cat No. 15596026). One milliliter of TRIZOL was added to harvested cells and mixed by pipetting up and down several times. To ensure efficient lysis, the mixture was vortexed for 15 s. The mixture was left at room temperature for 5 min to allow efficient dissociation of nucleic acid–protein complexes. Chloroform (Sigma, Cat. No. C2432) equivalent to 20% of the amount of TRIZOL used (200 µL) was added, vortexed for 15 sec, and left to settle for 3 min. The mixture was centrifuged at 12,000 × g for 15 min. After centrifugation, the top layer equivalent to ~50% of the volume of TRIZOL used (500 µL), was transferred to another tube. Volume equivalent to 50% of TRIZOL used (500 µL) of isopropanol (Sigma, Cat. No. I9516) was added, mixed, and left at room temperature for 10 min. The mixture was centrifuged at 12,000 × g for 10 min. The supernatant was removed, and the pellet washed with 1 mL of 75% ethanol. Centrifugation was done at 7500 × g for 5 min. The ethanol wash step was repeated, and the pellet was left to dry for 5 min on a benchtop. The RNA pellet was dissolved in RNase-free water (Santa Cruz, Cat. No. sc-204391).

### RT-qPCR

cDNA was synthesized using the iScript cDNA synthesis kit (Bio-Rad, Cat. No. 170-8891) following the manufacturer’s protocols. qPCR was performed using iTaq Universal SYBR Green Supermix (Bio-Rad, Cat. No. 172-5125) on a ThermoFisher Scientific QuantStudio 3 or 5 Real-Time PCR system. CT values were normalized to Actin and to control cells to achieve ΔΔCT values and expressed as relative expression. The primers used for qPCR are as follows:

**Table:** 

Gene	Primer sequence
*TRIP12* */TRIP12 CDS*	5-'CCCAACCACAAGACGACTCA-3'
	5'-ACTGCACTTTGGGTGCCTTA-3'
*TRIP12 3’UTR*	5'-CACCTGAGTCAAGGAAACATGTTACGCCTTC-3'
	5'-GCCTGATCACTTTAAGTCCATGGGGCC-3'
*ACTIN*	5'-CCAGATCATGTTTGAGACCTTCAAC-3'
	5'-CCAGAGGCGTACAGGGATAGC-3'
*CDH1*	5'-TTACTGCCCCCAGAGGATGA-3'
	5'-TGCAACGTCGTTACGAGTCA-3'
*CDH2*	5'-CCGGTTTCATTTGAGGGCAC-3'
	5'-TCCCTCAGGAACTGTCCCAT-3'
*CDH3*	5'-CTGTGCTGGGGGCTGTCCTGG-3'
	5'-GGAGGGGCTCCTTGATCTTCCGC-3'
*CLDN1*	5'-GAAGATGAGGATGGCTGTCATTGGGGG-3'
	5'-CGATTCTATTGCCATACCATGCTGTGGCAAC-3'
*OCLN*	5'-GAGTTGACAGTCCCATGGCATACTCTTCC-3'
	5'-GCTGCCTGAAGTCATCCACAGGCG-3'
*CRB3*	5'-CCACCAGCTCCAGCTCCGATGGC-3'
	5'-GGCAGCCAAGAGGGAGAAGACCACG-3'
*DSG3*	5'-GGGGCTCTTCCCCAGAACTACAGG-3'
	5'-CTCTATTCGCAATTCTCCATGAACCAATATGACC-3'
*DSC3*	5'-CTGCATCTGCTGCTGACCCTCGTG-3'
	5'-GCAGGCTTCACCAGCACGACTGAAG-3'
*KRT17*	5'-GCCCGTCTGGCTGCTGATGACTTCC-3'
	5'-CGCAGGCCATTGATGTCGGCCTCC-3'
*LAMB1*	5'-CCAGCGAATGTGCCCCTGTGGATGG-3'
	5'-GTGTTATGCCTGCACATGCAGTGTCCGTG-3'
*ITGB2*	5'-CTGCTCGCCCTGGTGGGGCTG-3'
	5'-CGGCAGCTGCTGACCTTGAACTTCGTG-3'
*ITGA5*	5'-GCCCCCGGGCTCCTTCTTCGG-3'
	5'-GGTGCTCCCACCAGCACACTGACC-3'
*SNAI1*	5'-TCTTTCCTCGTCAGGAAGCC-3'
	5'-GATCTCCGGAGGTGGGATGG-3'
*SNAI2*	5'-CTCCTCATCTTTGGGGCGAG-3'
	5'-CTTCAATGGCATGGGGGTCT-3'
*TWIST1*	5'-TCGGACAAGCTGAGCAAGATT-3'
	5'-GCAGCTTGCCATCTTGGAGT-3'
*TWIST2*	5'-GCAAGAAGTCGAGCGAAGAT-3'
	5'-GCTCTGCAGCTCCTCGAA-3'
*ZEB1*	5'-AGGATGACCTGCCAACAGAC-3'
	5'-CTTCAGGCCCCAGGATTTCTT-3'
*ZEB2*	5'-CCCTGGCACAACAACGAGAT-3'
	5'-AATTGCGGTCTGGATCGTGG-3'

### RNA sequencing and analysis

RNA was extracted as per above “RNA extraction” method and sent to Novogene (Singapore) for sequencing. The platform used is the HiSeq-PE150, and the library selection is “Eukaryotic Transcriptomic Library”. For RNA-seq analysis, RNA-seq paired reads were aligned to the human reference genome GRCh37/hg19 and the gencode release 19 transcript database using “STAR Aligner” (v2.5.1b)^58^ with default parameters. Transcript levels quantification were computed by ‘FeatureCounts’ from the “Rsubread” package (v1.24)^59^ with the parameter strand Specific set to 1. The transcript counts were subsequently subjected to differential expression analysis with “DESeq2” (v1.14.1)^60^ using a Likelihood Ratio Test to estimate the expression changes. Differentially expressed transcripts were determined as those with an adjusted p-value lower than 0.05 and an absolute Log2 fold change greater than one. The volcano plot of gene expression was drawn using the scatterplot function from R. Functional annotation in enriched pathways of the gene list was performed with ConsensusPathDB^61^, using the KEGG and REACTOME databases and *p*-adjusted values thresholds <0.01.

### Cell migration/ wound-healing assay

Cells were seeded in triplicates at 4 × 10^5^ cells per well in a 24-well plate in serum-free media with additives overnight. A wound was created using a 10 µL pipette tip by gently scratching the well from top to bottom in a vertical motion. Cells were washed four times, with 0.5 mL PBS to remove detached cells, and 2 mL of serum-free media with additives was added after the last wash. Live imaging of cells was performed using Zeiss Axio Z1 with a cell observer (Zeiss) with image capture every 0.5 h until wound closure. Quantification of wound area was performed using “ImageJ”. Polygon selection (“ImageJ”) was used to trace the boundary of bulk cells at a specified timepoint to measure the remaining wound area. Quantification of single-cell dislodgement was performed by counting the number of single-cells in images when the remaining wound area is 35–55% of the total image area.

### Single-cell tracking analysis

Single-cell tracking was performed using the ImageJ plugin, MTrackJ^62^, and reported previously^63^. Single-cell tracking and velocity measurement to study cellular migration using the MTrackJ plugin were also utilized and reported by others^64–66^. First, movie in avi file format was loaded onto ImageJ, and the MTrackJ plugin initiated. Cell tracking was performed by clicking the “add” icon in the MTrackJ plugin, and cells were tracked by selecting the single cells frame by frame to create track lines. Fifteen cells were tracked for each condition. To measure velocity, “scale” and “frame interval” options need to be set. To set scale, draw a line across the scale bar of indicated length (for example, 100 µm) in the image. Next, click “analyze” and “set scale” in ImageJ. The “distance in pixels” represents the line drawn. Input the “known distance” (100), and the “unit of length” (µm). In this case, 155 pixels represent 100 µm. To set frame interval, select “image” and then “properties.” Change the “frame interval” to the interval between each frame. In this case, it is 0.5 h. To measure velocity, once the tracks are added, click “measure” in the MTrackJ plugin. Velocity will be measured. Images and movies containing tracks were exported and saved from the MTrackJ plugin.

### Anoikis assay

Fifty microliters of 1% agarose was added into each well of a 96-well plate and left to solidify. Cells were counted, and 2 × 10^4^ cells in 100 µL were seeded per well in triplicates per condition. At timepoints 0 h and 24 h, 20 μL of CellTiter 96 Aqueous One Solution cell proliferation assay (MTS) (Promega, Cat. No. G3581) was added to each well. The plate was incubated at 37 °C, and the absorbance of each well was taken at 490 nm at each hour for a total of 3 h. The background absorbance in each well was normalized by subtracting the absorbance value of a well without cells to increase the signal-to-noise ratio. Twenty-four hours reading was normalized to 0 h reading and expressed as % viability.

### Immunofluorescence staining and imaging

Cells were seeded at 3 × 10^5^ cells per well in a 6-well plate and grown in a 5% CO_2_ incubator at 37 °C for 24 h. Cells were washed once with PBS and fixed in 3.7% (w/v) paraformaldehyde (Sigma, Cat. No. P6148) for 30 min. Following which, cells were washed thrice with PBS, permeabilized with 0.1% Triton-X (Naclai Tesque, Cat. No. 25987-85) for 10 min, washed thrice with PBS, blocked with 3% BSA containing 0.1 M glycine for 30 min, washed thrice with PBS, and incubated with primary antibody (1:500 in 3% BSA dissolved in PBS) for 1.5 h at room temperature or overnight at 4 °C. Fixed cells were washed thrice with PBS and incubated in Alexa Fluor 488 Goat anti-mouse IgG secondary antibody (Thermo Fisher, Cat. No. A-32723) (1:500 in 3% BSA dissolved in PBS) for 30 min at 37 °C. Cells were washed five times with Milli-Q water and stained with Hoechst 33342 (Santa Cruz, Cat. No. SC-200908) dye at 1 µg/mL in Mili-Q water for 30 min. Images were taken using a Zeis Axiovert.A1 microscope (Zeis).

### Statistical analysis

The paired tail student *t*-test was used for significance testing. *p* values are depicted as such (* *p* < 0.05; ** *p* < 0.01; *** *p* < 0.001). Where *p* > 0.05, the *p* value is shown to three decimal points. Variance is similar between groups. The number of independent replicates used for statistical analysis in all cases is indicated in the respective figure legends. *N* represents biological replicates. Error bars were plotted using either standard deviation (SD) or standard error of the mean (SEM) and are indicated in the respective figure legends.

## Supplementary information

Supplementary Movie 1

Supplementary Movie 2

Supplementary Movie 3

Supplementary Movie 4

Supplementary Movie 5

Supplementary Movie 6

Supplementary Movie 7

Supplementary Movie 8

Supplementary Movie 9

Supplementary Movie 10

Supplementary Movie 11

Supplementary Movie 12

Supplementary Figure legends

Supplementary Fig. 1

Supplementary Fig. 2

Supplementary Fig. 3

Supplementary Table 1. List of differentially regulated genes

## Data Availability

The datasets generated and/or analyzed during the current study are available from the corresponding author on reasonable request.
