# Supplementary material for: The oncogenic E3 ligase TRIP12 suppresses epithelial–mesenchymal transition (EMT) and mesenchymal traits through ZEB1/2
Source: Cell Death Discov. 2021 May 7;7:95. doi: 10.1038/s41420-021-00479-z (PMC8105346; doi:10.1038/s41420-021-00479-z)
Supplement: Supplementary file 13 — Supplementary Figure legends [file 41420_2021_479_MOESM13_ESM.docx]

**Supplementary Figure legends**

**The oncogenic E3 ligase TRIP12 suppresses epithelial-mesenchymal transition (EMT) and mesenchymal traits through ZEB1/2**

Kwok Kin Lee^1*^, Deepa Rajagopalan^1,2^, Shreshtha Sailesh Bhatia^1^, Roberto Tirado-Magallanes^1^, Wee Joo Chng^1,3,4^, Sudhakar Jha^1,2,*^

**Affiliations:**

^1^Cancer Science Institute of Singapore, National University of Singapore, Singapore 117599.

^2^Department of Biochemistry, Yong Loo Lin School of Medicine, National University of Singapore, Singapore.

^3^Department of Medicine, Yong Loo Lin School of Medicine, National University of Singapore, Singapore 117596.

^4^Department of Haematology-Oncology, National University Cancer Institute of Singapore, National University Health System

^*^To whom correspondence should be addressed:

Sudhakar Jha Ph.D., Centre for Translational Medicine, 14 Medical Drive, MD6-13-02J, Singapore 117599.

E-mail: [csisjha@nus.edu.sg](mailto:csisjha@nus.edu.sg), Phone: 65-66012402, Fax: 65-68739664

Kwok Kin Lee Ph.D., Centre for Translational Medicine, 14 Medical Drive, MD6-13-02S, Singapore 117599.

E-mail: csileek@nus.edu.sg, Phone: 65-65165993, Fax: 65-68739664

Running title: TRIP12 inhibits EMT through ZEB1/2

The authors declare no competing interests.

**List of supplementary figure legends**

**Supplementary Fig. 1.** Validation of EMT gene expression changes in breast cancer cell lines.

**Supplementary Fig. 2.** *TRIP12* expression level in MCF10A cells stably expressing ectopic TRIP12.

**Supplementary Fig. 3.** Anoikis assay and cell viability of MCF10A cells stably transfected with ectopic TRIP12.

**Supplementary Movie 1.** MCF10A shControl LHCX-vector, without cell tracking.

**Supplementary Movie 2.** MCF10A shTRIP12-2^(6427-6447)^ LHCX-vector, without cell tracking.

**Supplementary Movie 3.** MCF10A shControl LHCX-TRIP12, without cell tracking.

**Supplementary Movie 4.** MCF10A shTRIP12-2^(6427-6447)^ LHCX-TRIP12, without cell tracking.

**Supplementary Movie 5.** MCF10A shControl LHCX-vector, with cell tracking.

**Supplementary Movie 6.** MCF10A shTRIP12-2^(6427-6447)^ LHCX-vector, with cell tracking.

**Supplementary Movie 7.** MCF10A shControl LHCX-TRIP12, with cell tracking.

**Supplementary Movie 8.** MCF10A shTRIP12-2^(6427-6447)^ LHCX-TRIP12, with cell tracking.

**Supplementary Movie 9.** MCF10A shTRIP12-2^(6427-6447)^ siControl, without cell tracking.

**Supplementary Movie 10.** MCF10A shTRIP12-2^(6427-6447)^ siZEB1/2, without cell tracking.

**Supplementary Movie 11.** MCF10A shTRIP12-2^(6427-6447)^ siControl, with cell tracking.

**Supplementary Movie 12.** MCF10A shTRIP12-2^(6427-6447)^ siZEB1/2, with cell tracking.

**Supplementary Table 1.** List of differentially regulated genes

**Supplementary Fig. 1.** Validation of EMT gene expression changes in breast cancer cell lines. Relative expression of EMT markers mRNA levels in MDA-MB-468 (**a**) and CAL51 (**b**) stably depleted of TRIP12. Relative expression levels were normalized to Actin mRNA levels and data quantified relative to shControl. (N = 1). Data represents means ± SD.

**Supplementary Fig. 2.** *TRIP12* expression level in MCF10A cells stably expressing ectopic TRIP12. **a** Relative expression of *TRIP12* mRNA levels in MCF10A cells stably depleted of TRIP12 and with LHCX-vector expression or LHCX-TRIP12 expression using primers targeting TRIP12 CDS. Relative expression levels were normalized to Actin mRNA levels and data quantified relative to shControl LHCX-vector. (N = 3). Data represents means ± SEM. **b** TRIP12 and Actinin protein levels in MCF10A cells stably depleted of TRIP12 and with LHCX-vector expression or LHCX-TRIP12 expression. Actinin serves as a loading control. Arrow indicates the TRIP12 band. (N = 1).

**Supplementary Fig. 3.** Anoikis assay and cell viability of MCF10A cells stably transfected with ectopic TRIP12. **a** Percent cell viability data for MCF10A shControl and shTRIP12-2^(6427-6447)^ with either LHCX-vector or LHCX-TRIP12 expression seeded on agarose coated wells for 24 h. Cell viability was measured using a MTS assay and expressed as a percentage to 0 h. Data represents means ± SEM. (N = 3). **b** Percent cell viability data for MCF10A shControl and shTRIP12-2^(6427-6447)^ with either LHCX-vector or LHCX-TRIP12 expression seeded on normal tissue culture wells for 24 h. Cell viability was measured using a MTS assay and expressed as a percentage to 0 h. Data represents means ± SEM. (N = 2).

**Supplementary Movie Legends**

**Supplementary Movie 1.** MCF10A shControl LHCX-vector, without cell tracking. Movie showing the movement of MCF10A shControl LHCX-vector cells in a wound-healing assay. Images were taken at 10X magnification. Scale bar at bottom right corner = 100 µm.

**Supplementary Movie 2.** MCF10A shTRIP12-2^(6427-6447)^ LHCX-vector, without cell tracking. Movie showing the movement of MCF10A shTRIP12-2^(6427-6447)^ LHCX-vector cells in a wound-healing assay. Images were taken at 10X magnification. Scale bar at bottom right corner = 100 µm.

**Supplementary Movie 3.** MCF10A shControl LHCX-TRIP12, without cell tracking. Movie showing the movement of MCF10A shControl LHCX-TRIP12 cells in a wound-healing assay. Images were taken at 10X magnification. Scale bar at bottom right corner = 100 µm.

**Supplementary Movie 4.** MCF10A shTRIP12-2^(6427-6447)^ LHCX-TRIP12, without cell tracking. Movie showing the movement of MCF10A shTRIP12-2^(6427-6447)^ LHCX-TRIP12 cells in a wound-healing assay. Images were taken at 10X magnification. Scale bar at bottom right corner = 100 µm.

**Supplementary Movie 5.** MCF10A shControl LHCX-vector, with cell tracking. Movie showing the movement of MCF10A shControl LHCX-vector cells in a wound-healing assay. Images were taken at 10X magnification. Scale bar at bottom right corner = 100 µm.

**Supplementary Movie 6.** MCF10A shTRIP12-2^(6427-6447)^ LHCX-vector, with cell tracking. Movie showing the movement of MCF10A shTRIP12-2^(6427-6447)^ LHCX-vector cells in a wound-healing assay. Images were taken at 10X magnification. Scale bar at bottom right corner = 100 µm.

**Supplementary Movie 7.** MCF10A shControl LHCX-TRIP12, with cell tracking. Movie showing the movement of MCF10A shControl LHCX-TRIP12 cells in a wound-healing assay. Images were taken at 10X magnification. Scale bar at bottom right corner = 100 µm.

**Supplementary Movie 8.** MCF10A shTRIP12-2^(6427-6447)^ LHCX-TRIP12, with cell tracking. Movie showing the movement of MCF10A shTRIP12-2^(6427-6447)^ LHCX-TRIP12 cells in a wound-healing assay. Images were taken at 10X magnification. Scale bar at bottom right corner = 100 µm.

**Supplementary Movie 9.** MCF10A shTRIP12-2^(6427-6447)^ siControl, without cell tracking. Movie showing the movement of MCF10A shTRIP12-2^(6427-6447)^ siControl cells in a wound-healing assay. Images were taken at 10X magnification. Scale bar at bottom right corner = 100 µm.

**Supplementary Movie 10.** MCF10A shTRIP12-2^(6427-6447)^ siZEB1/2, without cell tracking. Movie showing the movement of MCF10A shTRIP12-2^(6427-6447)^ siZEB1/2 cells in a wound-healing assay. Images were taken at 10X magnification. Scale bar at bottom right corner = 100 µm.

**Supplementary Movie 11.** MCF10A shTRIP12-2^(6427-6447)^ siControl, with cell tracking. Movie showing the movement of MCF10A shTRIP12-2^(6427-6447)^ siControl cells in a wound-healing assay. Images were taken at 10X magnification. Scale bar at bottom right corner = 100 µm.

**Supplementary Movie 12.** MCF10A shTRIP12-2^(6427-6447)^ siZEB1/2, with cell tracking. Movie showing the movement of MCF10A shTRIP12-2^(6427-6447)^ siZEB1/2 cells in a wound-healing assay. Images were taken at 10X magnification. Scale bar at bottom right corner = 100 µm.

**Supplementary Table Legends**

**Supplementary Table 1.** List of differentially regulated genes
