# Supplementary figures and images for: The oncogenic E3 ligase TRIP12 suppresses epithelial–mesenchymal transition (EMT) and mesenchymal traits through ZEB1/2
Source: Cell Death Discov. 2021 May 7;7:95. doi: 10.1038/s41420-021-00479-z (PMC8105346; doi:10.1038/s41420-021-00479-z)

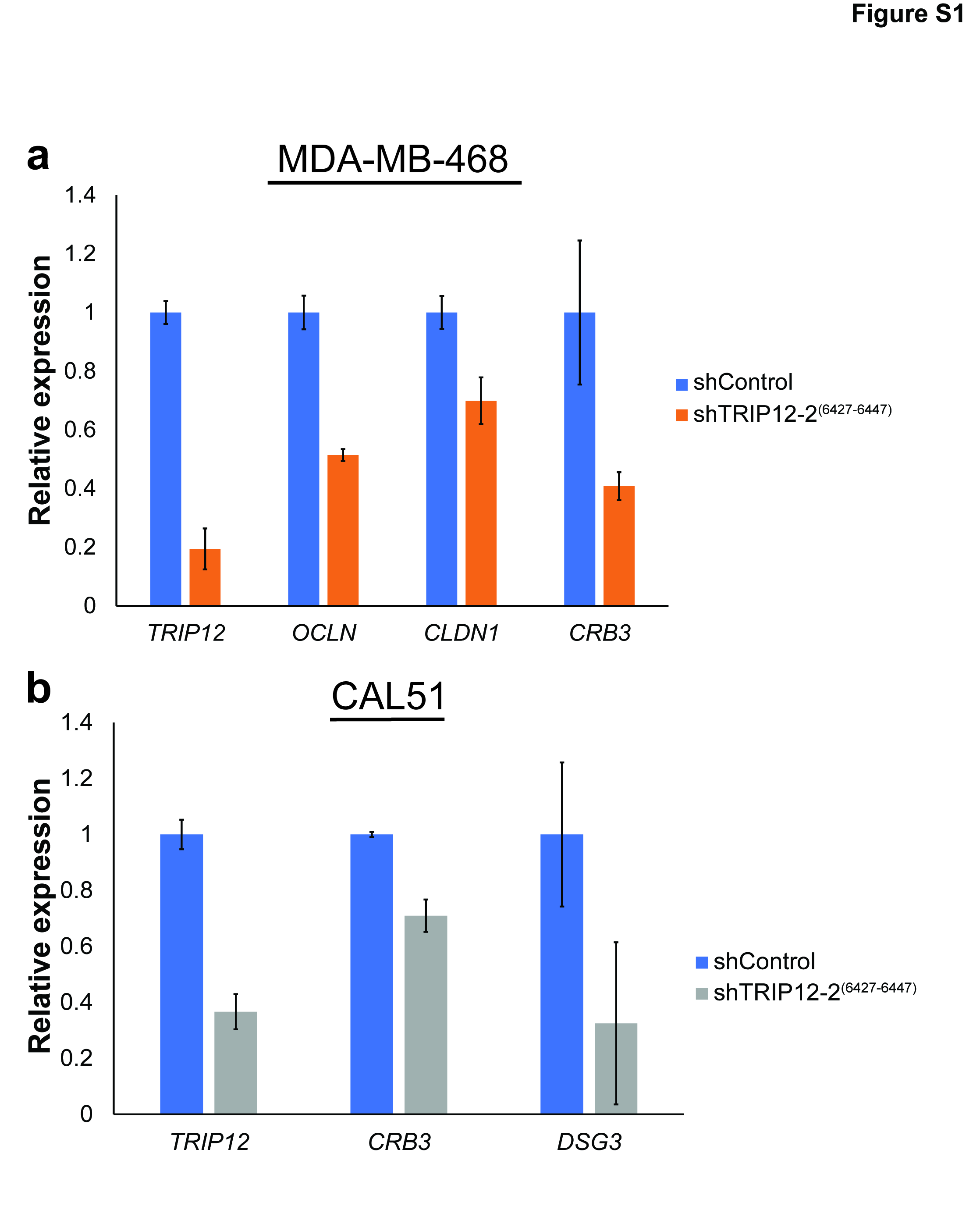

Supplement: Supplementary file 14 — Supplementary Fig. 1 [file 41420_2021_479_MOESM14_ESM.tif]

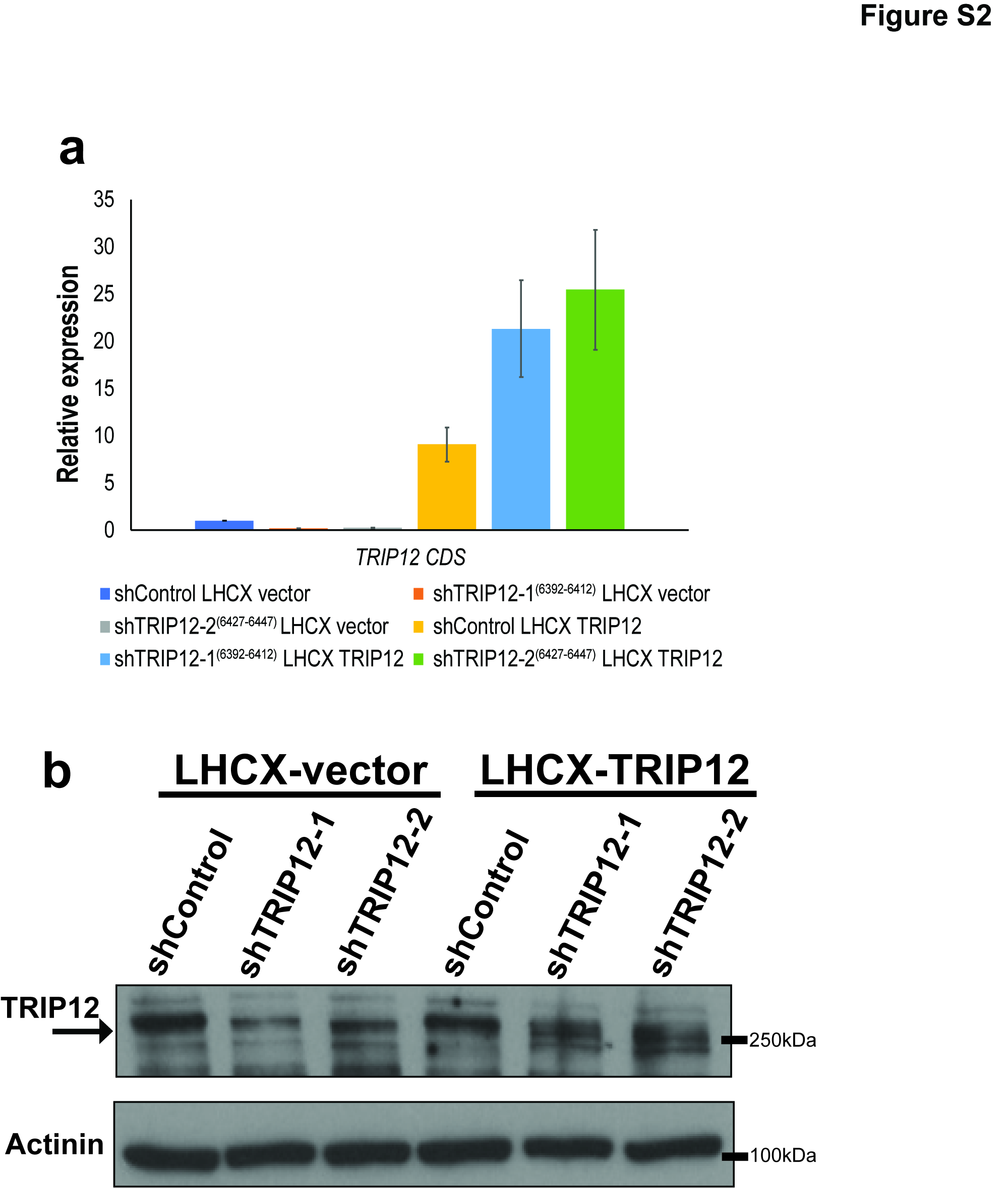

Supplement: Supplementary file 15 — Supplementary Fig. 2 [file 41420_2021_479_MOESM15_ESM.tif]

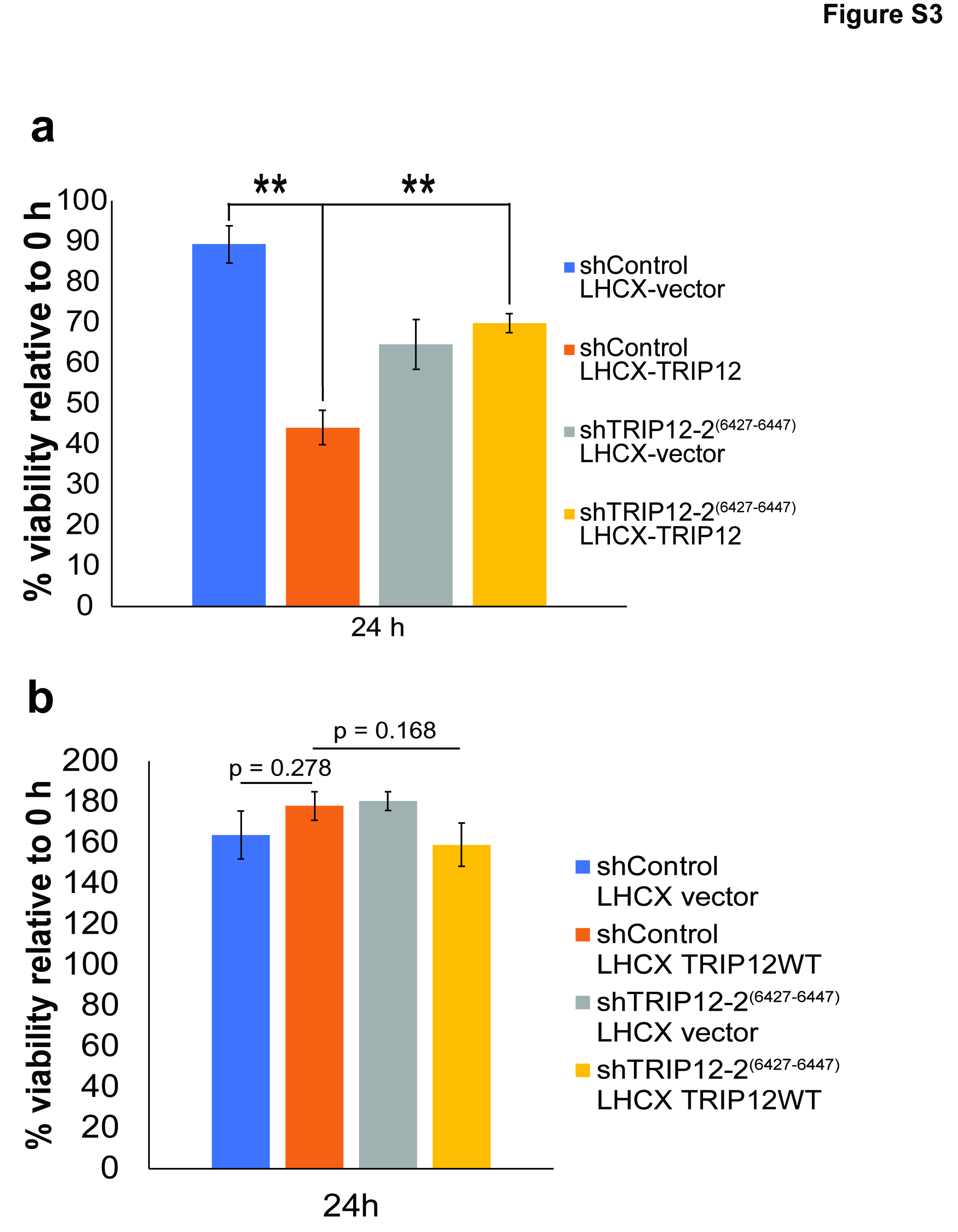

Supplement: Supplementary file 16 — Supplementary Fig. 3 [file 41420_2021_479_MOESM16_ESM.tif]
